# Editorial: Virtual, mixed, and augmented reality in cognitive neuroscience and neuropsychology

**DOI:** 10.3389/fpsyg.2022.1010852

**Published:** 2022-09-02

**Authors:** Dalila Burin, Adriana Salatino, Mounia Ziat

**Affiliations:** ^1^Smart-Aging Research Centre (SARC), Institute of Development, Aging and Cancer (IDAC), Tohoku University, Sendai, Japan; ^2^Institute of Neuroscience, Université Catholique de Louvain Brussels, Brussels, Belgium; ^3^Department of Psychology, University of Turin, Turin, Italy; ^4^Information Design and Corporate Communication, Bentley University, Waltham, MA, United States

**Keywords:** virtual reality, augmented reality, cognitive neuroscience, neuropsychology, ownership, sense of agency, visuo-spatial disorders, embodied cognition

## Introduction

Among many endeavors in scientific research, virtual reality (VR) is one of the most compelling and fruitful scenario for cognitive neuroscience and neuropsychology (Bohil et al., 2011; Parsons et al., 2020). Because of its flexibility and adaptability to different scopes, VR technology has advanced from mere display-VR; where the simulation is implemented on a 2D monitor with depthless interaction, to immersive virtual reality (IVR), that mimics and overlaps with the physical world that fully engages the physical body (Slater, 2009).

VR allows the full manipulation of environmental (such as visual scenarios) and body-related (such as somatic features) parameters (Slater et al., 2008; Peck et al., 2013; Chan et al., 2021); as additional advantage, it can be used in combination with other measurements, such as brain-computer interface (BCI) (Nierula et al., 2021) and motion tracking systems (Banakou et al., 2013), making it a noteworthy method for neuroscientists to explore motor and cognitive functions.

While the initial usage of VR in cognitive neuroscience was to provide a sense of presence by properly representing the virtual setting and the virtual body (Sanchez-Vives and Slater, 2005), it has more recently expanded into diagnostic and rehabilitation strategies for clinical populations (Matamala-Gomez et al., 2021). Several studies involving VR ranges from the exploration of basic concepts such as the motor and bodily self-consciousness (Herbelin et al., 2016), to clinical treatments (Ziat et al., 2014; Chin et al., 2021) and approaches for attention and visuo-spatial disorders (Gammeri et al., 2020; De Luca et al., 2021) and clinical.

## Research topic content

The aim of this Research Topic “*Virtual, Mixed, and Augmented Reality in Cognitive Neuroscience and Neuropsychology*” published in Frontiers in Psychology-Neuropsychology, was to highlight the potential of Virtual Reality into developing new tools for research in cognitive psychology and clinical research. The Research Topic features 7 research articles. Most of the contributions examined spatial disorientation, memory distortion, and multisensory effects on attention. Based on their contributions, the research articles were grouped into three main areas: somatic and spatial features of the virtual body (2 papers), Assessing Learning and Memory in VR (2 papers), IVR Assessment of Attentional and Spatial Disorders (3 papers).

### Somatic and spatial features of the virtual body

To enhance immersion, the displayed virtual body, known as avatar, must comprise certain characteristics. By combing BCI and VR, Ziadeh et al. measured the sense of body ownership and agency during a motor imagery task by comparing two levels of embodiments: a humanoid virtual hand to induce a strong level of embodiment, and abstract blocks to infer a low level of embodiment. It was shown that the ownership over the humanoid hand led to an increased sense of agency, when controlling for performance. The authors recommended the implementation of anthropomorphic visual feedback on the virtual body, instead of using abstract images, in order to increase the body embodiment. The position of the avatar plays a critical role in the VR interaction. del Aguila et al. looked for the optimal interpersonal distance (IPD) between the avatar and the agent during a facial emotion recognition task. The IPD is an important concept in virtual social interaction as it determines the maintained distance between the avatar and the agent without invading the personal space of the agent. While general emotion recognition in IVR was very effective, all tested IPDs were considered equal in terms of emotional recognition. For specific emotions such as anger and sadness, a 75 cm distance provided the best results.

### Assessing learning and memory in VR

Navigation is a key feature in VR that can offer major benefits in studying memory and learning processes in both healthy individuals and neurological patients. Daviddi et al. focused on how mnemonic reactivation can be either enhanced or distorted by visual exposure and vividness of memory by asking healthy subjects to freely explore artworks in a virtual tour of the Louver museum. This encoding phase was followed by a reactivation phase, 2 h later, and a recognition phase, about approximately 24 h later, where participants were asked to recall old and new artworks (lures). The reactivation phase led to high recognition rate of the reactivated targets (previously seen artworks), but also a high false alarm yet where lures were recalled as being part of the virtual tour. This result is consistent with the premise that memory reactivation is an active process. During the recognition phase, higher living rates were highly correlated with viewing time. Old lures (presented during the reactivation phase) had high living rates comparing to new lures. Visitors vividly believed they were part of the initial virtual museum tour suggesting that memory intrusions are more easily incorporated into true memories.

Virtual Learning and Training is getting a lot of attention. In a clinical setting, Biffi et al. used an IVR where children with cerebral palsy (CB) and typical developing (TD) peers had to navigate in a 5-way maze in a playground to find a reward. While typical participants immediately succeeded the task, independently from the strategy they preferred, half of the children with CP needed more attempts with longer time to identify the best navigation method. They also had a preference for egocentric strategies based on body motion comparing to TD children who mainly relied on allocentric strategies based on environmental cues. The efficiency of the dynamic process of learning and adaptation to environmental modifications seem to be the crucial point for children with CP patients. IVR controlled environments can support this adaptability and help with learning.

### IVR assessment of attentional and spatial disorders

A very commonly studied disorders in VR is unilateral spatial neglect (USN) that consists of the failure to report, respond, or orient to stimuli presented to opposite side of the lesioned hemisphere, when this failure cannot be attributed to either sensory or motor defects (Heilman et al., 2000). In their systematic review, Kaiser et al. highlighted the efficiency of combination of IVR and eye tracking (ET) as a tool to design tasks and measures for USN patients and help identify those undiagnosed with traditional tests. IVR specifically guarantees a high level of ecological validity, while ET, in particular, can potentially be useful in differentiating subtypes of USN. A practical example comes from Hougaard et al.'s study where they compare the IVR-ET system with conventional test for a USN diagnosis. IVR-ET combination was more sensitive in detecting three egocentric neglect patients that conventional tests failed to detect. Contrariwise, four patients, that were not detected in the VR system, were picked by the traditional tests. This work emphasizes the importance of complementary diagnostics tools, such as IVR-ET as spatial neglect symptoms are highly heterogeneous. Additional testing and assessment programs can benefit from IVR. Dozio et al. proposed an IVR version of the Bell Test (Gauthier et al., 1989), an established neuropsychological test to assess attentional and visuospatial disorders, to investigate the effect of smell (peppermint scent), and audition (piano tones) on visuospatial attention. When single or multisensory stimulations came from the left side, the performance improved; results that can be explained by the pseudoneglect phenomenon (Jewell and McCourt, 2000), where adults tend to overestimate the left side of the visual hemispace. When both stimuli came from the right side, they were significantly more effective in capturing participants' visuospatial attention. This study emphasizes the importance of multisensory stimulation and provides interesting insights about multisensory combination for diagnosis and treatment of neuropsychological disorders, such as USN.

## Outlook

In this Research Topic, researchers described a wide range of challenges in using VR in laboratory and clinical settings, ranging from the importance to the avatar features to the importance of using more IVR environments for assessments, scenarios, and treatments. VR can also be an effective investigation tool for understanding cognitive functions such as learning and memory. The evidence produced by this collection of papers collectively highlights the growing trend for using VR in neuropsychology.

## Author contributions

All authors listed have made a substantial, direct, and intellectual contribution to the work and approved it for publication.

## Conflict of interest

The authors declare that the research was conducted in the absence of any commercial or financial relationships that could be construed as a potential conflict of interest. The handling editor SP declared a shared affiliation with the author AS at the time of review.

## Publisher's note

All claims expressed in this article are solely those of the authors and do not necessarily represent those of their affiliated organizations, or those of the publisher, the editors and the reviewers. Any product that may be evaluated in this article, or claim that may be made by its manufacturer, is not guaranteed or endorsed by the publisher.
